# Genomic Identification and Comparative Characterization of Chemosensory Genes in Two Walnut Pests

**DOI:** 10.3390/biology15171471

**Published:** 2026-08-31

**Authors:** Dan-Dan Feng, Jia-Tong Liu, Cai-Qing Yang, Ai-Bing Zhang

**Affiliations:** College of Life Sciences, Capital Normal University, Beijing 100048, China

**Keywords:** *Conogethes punctiferalis*, *Atrijuglans aristata*, odorant receptor (OR), gustatory receptor (GR), odorant-binding protein (OBP), chemosensory protein (CSP), genome annotation, phylogeny

## Abstract

*Conogethes punctiferalis* (generalist) and *Atrijuglans aristata* (specialist) are important pests of walnut fruits. Chemosensory genes play critical roles in host location and mating, making them promising candidates for pest management research. However, genome-wide identification of these gene families has not yet been performed for either species, and cross-species comparisons are often confounded by differences in annotation quality and methodology. Here, we employed a unified pipeline for genome annotation and gene family identification to systematically characterize the odorant receptor (OR), gustatory receptor (GR), odorant-binding protein (OBP), and chemosensory protein (CSP) genes of both species and further analyzed their physicochemical properties, chromosomal distribution, and phylogeny. Genome annotation identified 13,236 and 14,083 protein-coding genes in *C. punctiferalis* and *A. aristata*, respectively, with BUSCO completeness of 94.6% and 94.5%. We identified 121 candidate chemosensory genes in *C. punctiferalis* (46 ORs, 23 GRs, 32 OBPs, and 20 CSPs) and 137 in *A. aristata* (65 ORs, 26 GRs, 28 OBPs, and 18 CSPs). Notably, *A. aristata* possesses more OR genes (65) than *C. punctiferalis* (46). Both species possess a single conserved ORco, with three and four pheromone receptor (PR) genes in *C. punctiferalis* and *A. aristata*, respectively. Chemosensory genes were either dispersed or tandemly arrayed on chromosomes, with each family clustering into conserved functional branches. This study provides a reliable foundation for comparative chemosensory evolution studies and a catalog of candidate genes for future functional validation.

## Simple Summary

*Conogethes punctiferalis* and *Atrijuglans aristata* are two major insect pests infesting walnut fruits in China. The former is a generalist, whereas the latter is a specialist. Chemosensory genes regulate a broad spectrum of physiological and behavioral processes in insects, rendering these genes promising candidates for functional studies related to pest management. In this study, we employed a standardized pipeline for genome annotation and gene family identification to systematically identify and compare the chemosensory gene families of both species. Based on these standardized annotations, our results reveal that the two species possess similar numbers of candidate gustatory receptor (GR), odorant-binding protein (OBP), and chemosensory protein (CSP) genes, whereas *A. aristata* harbors more candidate odorant receptor genes (65) than *C. punctiferalis* (46). The identified chemosensory genes were either tandemly arrayed or dispersed on chromosomes. Our findings provide valuable genomic resources for the two walnut pest species and establish a catalog of candidate chemosensory genes for future functional validation.

## 1. Introduction

Common walnut (*Juglans regia*) is an economically important tree species in China, cultivated nationwide for its nutrient-dense nuts [1,2,3]. China produced approximately 1.55 million metric tons of walnuts in 2025/2026, ranking first in the world (https://www.statista.com/statistics/675974/walnut-production-worldwide-by-country/, accessed on 13 June 2026). In recent years, however, walnut production has been severely compromised by two major pests: *Conogethes punctiferalis* and *Atrijuglans aristata* (syn. *A. hetaohei*) [4]. The former is a polyphagous insect that feeds on walnut as well as over 40 other species of fruits, field crops, and forest trees [5]. The latter is a specialist pest that feeds primarily on cultivated walnut fruits, with only occasional records on *Juglans mandshurica* [4,6]. These two species represent the primary biotic constraints to walnut production and have caused substantial economic losses. Infestation rates have been reported to reach 40–50% for *A. aristata* and 4–16% for *C. punctiferalis* [7,8]. Since 2018, however, *C. punctiferalis* has overtaken *A. aristata* as the dominant insect pest of walnut fruits [4]. The persistent damage caused by these two pests has severely hindered the sustainable development of the walnut industry and underscores the urgent need for further research on these insects to guide the development of environmentally sound management strategies.

Chemosensory mechanisms play critical roles in many insect behaviors, including host plant location, mate finding, and predator avoidance [9]. These processes typically involve the coordinated action of multiple gene families, such as odorant receptors (ORs), gustatory receptors (GRs), odorant-binding proteins (OBPs), and chemosensory proteins (CSPs) [10]. As soluble proteins, OBPs and CSPs serve as the first stage of processing in the olfactory system, responsible for transporting pheromones and general odorant molecules to the dendritic membrane surfaces of olfactory sensory neurons [11]. OBPs can be classified into pheromone-binding proteins (PBPs) and general odorant-binding proteins (GOBPs), along with other types such as antennal-binding proteins (ABPs) [12]. Beyond their essential functions in olfactory recognition, these proteins also play significant roles in mediating insecticide resistance [13]. CSPs are highly conserved among insect species but display considerable functional versatility. In addition to olfaction, they also participate in a range of physiological functions, such as tissue repair and morphogenesis [14]. Both ORs and GRs are membrane-bound receptors with seven transmembrane domains. ORs form heteromeric complexes with the conserved co-receptor ORco; these complexes function as ligand-gated cation channels that recognize odorants, transduce chemical signals into electrical responses, and ultimately trigger behavioral outputs [14,15]. Some members of the OR family have specialized as pheromone receptors (PRs), which specifically recognize sex pheromones released by individuals of the opposite sex and play critical roles in mating behavior [16]. Previous evidence indicates that ORs originated from the GR family [17]. GRs are located in gustatory receptor neurons of the taste organs and are classified into sugar, fructose, CO_2_, and bitter receptors based on their ligand selectivity [18]. Given their involvement in diverse essential physiological and behavioral functions, chemosensory genes have become a major focus in the development of novel pest management strategies [19].

The sizes of chemosensory gene families often vary among insects with different dietary breadths, with polyphagous species tending to possess larger gene families than specialists, especially in the GR family [20]. However, different gene annotation methods can yield substantially different orthology inferences, thereby introducing bias in cross-species comparisons of gene family numbers [21]. For instance, the number of lineage-specific genes identified can differ by as much as 15-fold among different annotation approaches [22]. Moreover, data types and alternative splicing also affect the completeness of gene identification [23,24]. Therefore, when conducting cross-species comparative analyses, it is essential to use consistent data types, especially genome-wide data, and a unified analytical pipeline for gene family identification, so that gene family sizes can be compared with minimal methodological bias.

Although chromosome-level, high-quality genome assemblies have been reported for *C. punctiferalis* and *A. aristata* [25,26], and chemosensory-related genes have been preliminarily identified using transcriptomic data, with functional validation conducted for a small number of genes [27,28,29], a systematic investigation at the whole-genome level remains lacking. Moreover, the differences in chemosensory gene family sizes between these two walnut pests have not yet been compared. To comprehensively characterize the chemosensory gene features of these two walnut pests, we first performed genome annotation using a standardized pipeline based on high-quality genomes. On this basis, we systematically identified four gene families—ORs, GRs, OBPs, and CSPs—and characterized their physicochemical properties, chromosomal distribution, and phylogenetic relationships. This study provides a descriptive comparison of the chemosensory gene repertoires of two walnut pests and establishes a foundation for future comparative, evolutionary, and functional studies.

## 2. Materials and Methods

### 2.1. Genome Acquisition, Annotation, and Completeness Assessment

Genome assembly data for *C. punctiferalis* and *A. aristata* were downloaded from NCBI under accession numbers GCA_031163375.1 [26] and GCA_046579625.1 [25]. Transcriptome data for *C. punctiferalis* were obtained from SRA under accession numbers SRR25435727–SRR25435730 and SRR35045396, while those for *A. aristata* were derived from all larval transcriptome datasets: SRR23462686, SRR31891378, and SRR31891379. Detailed information for these transcriptome datasets is provided in Supplementary Table S1. Gene annotation was performed using the NCBI Eukaryotic Genome Annotation Pipeline-external (EGAPx v0.2-alpha; https://github.com/ncbi/egapx, accessed on 4 November 2024). In the configuration file, we specified the full paths to the genome assembly and transcriptome sequencing reads and set the species taxonomic ID according to the NCBI taxonomy database. All other parameters were kept at their default settings. To ensure the reliability of the resulting gene sets, completeness was assessed using BUSCO v5.4.7 [30] in protein mode against the insecta_odb10 dataset (dataset date 10 September 2020), comprising 1367 single-copy orthologs, with all other parameters kept at their default settings. In addition, OMArk v0.4.1 [31] was used to evaluate both the completeness and consistency of the annotated gene sets. Briefly, OMAmer v2.1.2 [32] and OMArk were run with default parameters against the LUCA database (OMAmerDB; constructed from the December 2021 release of the OMA database), and completeness and consistency were assessed at the ancestral lineage Obtectomera.

### 2.2. Identification of Chemosensory Gene Families in the Two Species

To avoid redundant bias introduced by alternatively spliced isoforms in subsequent analyses and to ensure that each gene locus was represented by a single non-redundant sequence, only the longest transcript per gene was retained as the representative sequence for downstream gene family identification. Given that insect chemosensory gene families generally evolve rapidly and exhibit low sequence similarity across species [33], we employed two complementary approaches, homology search and domain-based screening, to identify members of each chemosensory gene family in the two species. For the homology search approach, we first performed searches against the NCBI database using “Lepidoptera” and the name of each gene family as keywords and retrieved the resulting sequences (retrieved in June 2026). These sequences were then filtered by removing gene IDs containing keywords such as “LOW QUALITY,” “PREDICTED,” “uncharacterized,” “partial,” “putative,” and “hypothetical,” as well as those with unrelated terms in their IDs, to retain high-confidence chemosensory gene sequences; the retained sequences were designated as the clean reference set. A local BLAST database was subsequently constructed from the clean data using makeblastdb (BLAST+ v2.14.1+), and the amino acid sequences of both species were used as queries in BLASTP searches against this database with an *e*-value threshold of 1 × 10^5^; sequences with significant hits were retained. For the domain-based screening approach, Hidden Markov model (HMM) profiles corresponding to the characteristic domains of each gene family were downloaded from the Pfam database: ORs (PF02949 or PF13853), GRs (PF06151 or PF08395), OBPs (PF01395), and CSPs (PF03392). Domain searches were performed using hmmsearch from HMMER v3.3.2 [34] under the same *e*-value cutoff. Genes identified by both approaches were retained as initial candidates of each gene family.

### 2.3. Physicochemical Characterization and Chromosomal Localization of Chemosensory Genes

Open reading frames (ORFs) of all chemosensory genes were manually verified. Transmembrane domains (TMDs) of ORs and GRs were analyzed using TMHMM-2.0 [35], while signal peptides of OBPs and CSPs were predicted using SignalP 6.0 [36] under the Eukarya model. Additional parameters, including molecular weight (MW), isoelectric point (pI), instability index (II), aliphatic index (AI), and grand average of hydropathicity (GRAVY), were calculated using the ProteinAnalysis module of Biopython v1.85 [37].

Conserved motif prediction of CSP protein sequences was performed using MEME software [38], with the motif occurrence mode set to ZOPS (zero or one occurrence per sequence). Parameters were configured as follows: maximum number of motifs to discover set to 10, motif width ranging from 6 to 50 amino acids, classic objective function, and 0-order Markov model as the background model.

Based on the criteria described above, candidate chemosensory genes were further classified as follows: Genes were considered putative intact genes when they contained a complete ORF without premature stop codons, retained the start codon, exhibited protein lengths comparable to those of their closest homologs, and possessed the family-specific core domains, such as signal peptides in OBPs/CSPs and transmembrane domains in ORs/GRs. Genes whose coding regions contained premature stop codons were classified as putative pseudogenes. Genes that were markedly shorter than their closest homologs, lacked the core domains, or lacked a predicted signal peptide (in the case of secreted protein families) were classified as putative incomplete genes. Genes that did not fall into any of the above categories were tentatively designated as putative fused genes. Pseudogenes were excluded from gene counting and subsequent analyses.

To further investigate the chromosomal distribution patterns of the identified candidate chemosensory genes, their genomic positions were extracted from the genome annotation files (GFF) and visualized using custom Python v3.10.18 scripts implemented with matplotlib v3.10.3 [39].

### 2.4. Phylogenetic Analysis

Phylogenetic trees were constructed using amino acid sequences of candidate OR, GR, and OBP genes from *C. punctiferalis* and *A. aristata*, together with homologs from representative lepidopteran species. Sequences were aligned using MUSCLE v3.8.1551 [40], and poorly aligned regions were trimmed using trimAl v1.4.rev15 [41] with the automated1 mode. Trimming reduced the OR, GR, and OBP alignments from 2805, 1259, and 857 positions to 264, 267, and 157 positions, respectively. Maximum likelihood trees were conducted using IQ-TREE v2.3.6 [42]; the best-fit substitution models were selected by the built-in ModelFinder [43], and branch support was evaluated using Ultrafast Bootstrap (UFBoot2) [44] with 1000 replicates. The best-fit models for the three gene families were as follows: OR, VT+F+R8; GR, VT+F+R8; OBP, LG+R4. Given that insect OR genes are thought to have evolved from the GR gene family and that OBPs and CSPs exhibit similarities in both physicochemical properties and functional roles [16,45,46], we selected one GR, one OR, and one CSP gene from *A. aristata* as outgroups to root the OR, GR, and OBP trees, respectively. To assess the expression levels of the identified chemosensory genes, RNA-seq reads from *C. punctiferalis* female antennae (SRR3194010) and *A. aristata* female heads (SRR10242502) were downloaded from the NCBI SRA database. Reads were aligned to the respective reference genomes using HISAT2 v2.1.0 [47], assembled with StringTie v2.2.1 [48], and quantified with featureCounts v2.0.6 [49]. The resulting phylogenetic trees and expression patterns were visualized using tvBOT [50].

## 3. Results

### 3.1. Genome Annotation Statistics of the Two Species

To eliminate the influence of different genome annotation methods on gene family identification, we re-annotated the genomes of *C. punctiferalis* and *A. aristata* using the same pipeline. A total of 13,236 and 14,083 protein-coding genes were annotated in *C. punctiferalis* and *A. aristata*, with average gene lengths of 24,234.7 bp and 21,951.9 bp, respectively (Table 1). Under the regulation of alternative splicing, 19,272 and 22,461 transcripts were annotated in the two species, respectively. Furthermore, more than 99% of genes in both species possessed complete ORFs. BUSCO assessment revealed that the completeness of structural annotation was 94.6% for *C. punctiferalis* and 94.5% for *A. aristata*. In contrast to BUSCO, OMArk not only evaluates the completeness of genome annotations by aligning query protein sequences against conserved genes in the Hierarchical Orthologous Groups (HOGs) of the ancestral lineage but also assesses their consistency by comparison with known gene families of this lineage and further enables the identification of potential contamination events in the genome [31]. Our OMArk results revealed that the completeness of genome annotation for *C. punctiferalis* was 92.48%, based on conserved Hierarchical Orthologous Groups (HOGs) within the ancestral lineage Obtectomera, with 88.22% single-copy and 4.26% duplicated genes. In terms of consistency, 89.76% of the annotated proteins were correctly assigned to known gene families of Obtectomera, while 4.29% were classified as inconsistent and 5.95% as unknown. Furthermore, no significant contamination events were detected across the entire proteome. The completeness and consistency scores for *A. aristata* were 92.84% and 89.17%, respectively, comparable to those of *C. punctiferalis*. Collectively, these results indicate that the genome annotations of both species are suitable for downstream gene family identification.

### 3.2. Identification of Chemosensory Gene Families

**Figure 1:**
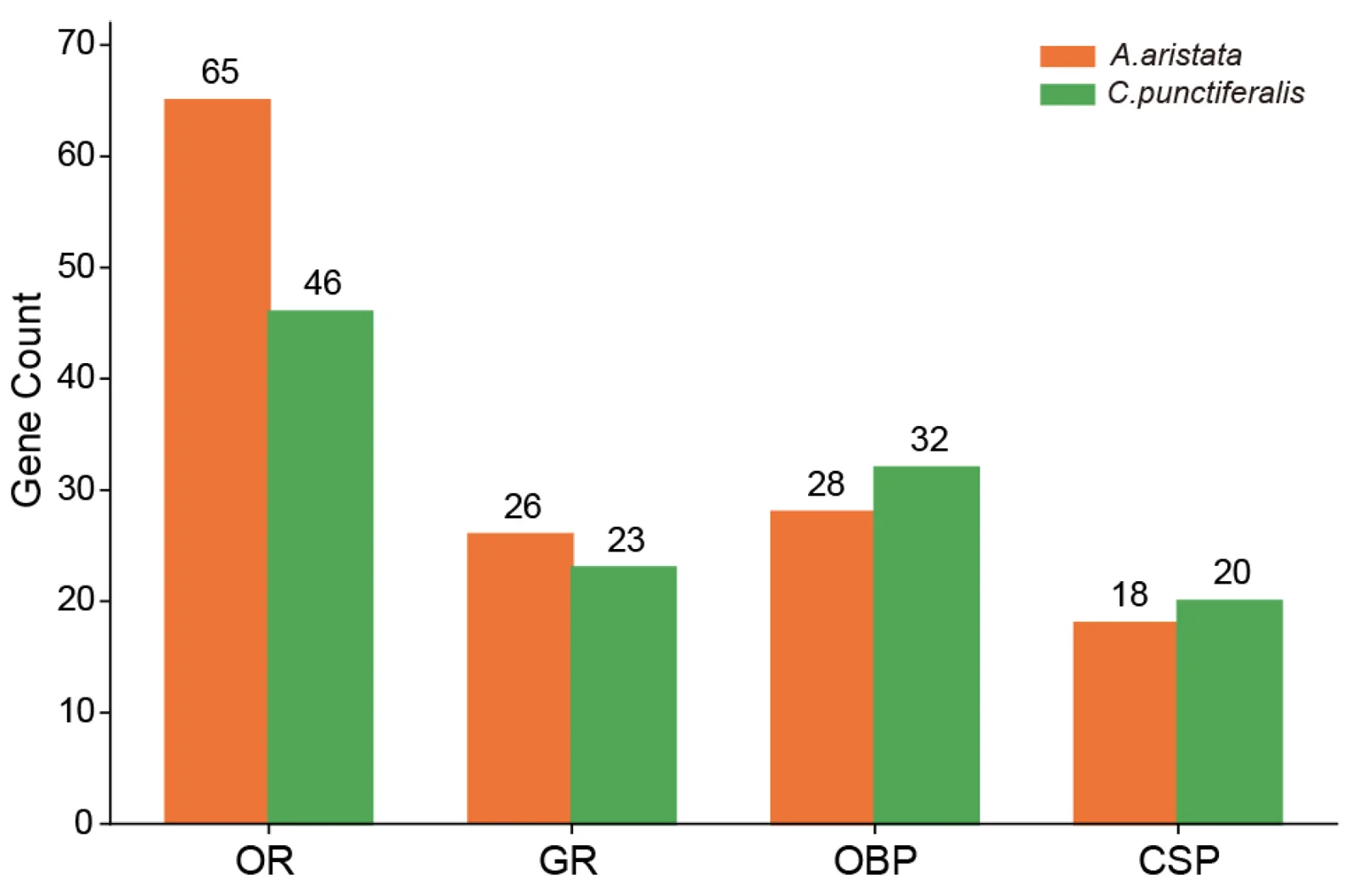
Comparison of chemosensory gene family sizes between *Conogethes punctiferalis* and *Atrijuglans aristata*.

Based on the standardized annotation results, we employed two complementary approaches—homology search and domain screening—to cross-validate the identification of chemosensory gene families in the genomes of *C. punctiferalis* and *A. aristata* (Supplementary Table S2). In *C. punctiferalis*, a total of 122 putative chemosensory-related genes were identified, including one pseudogene, *CpunOR74*; the remaining 121 genes comprised 46 ORs, 23 GRs, 32 OBPs, and 20 CSPs (Figure 1). In *A. aristata*, 137 candidates were identified, including 65 ORs, 26 GRs, 28 OBPs, and 18 CSPs. Specifically, *C. punctiferalis* possessed only four more OBP genes and two more CSP genes than *A. aristata*, while its GR genes were even three copies fewer. In contrast, *A. aristata* possessed more OR genes (65) than *C. punctiferalis* (46). Meanwhile, we also found that these genes in *C. punctiferalis* exhibited one-to-one or one-to-many correspondence with the OR genes reported by Ge et al. [27] (Supplementary Table S3).

### 3.3. Feature Analysis and Chromosomal Localization of Chemosensory Genes

**Figure 2:**
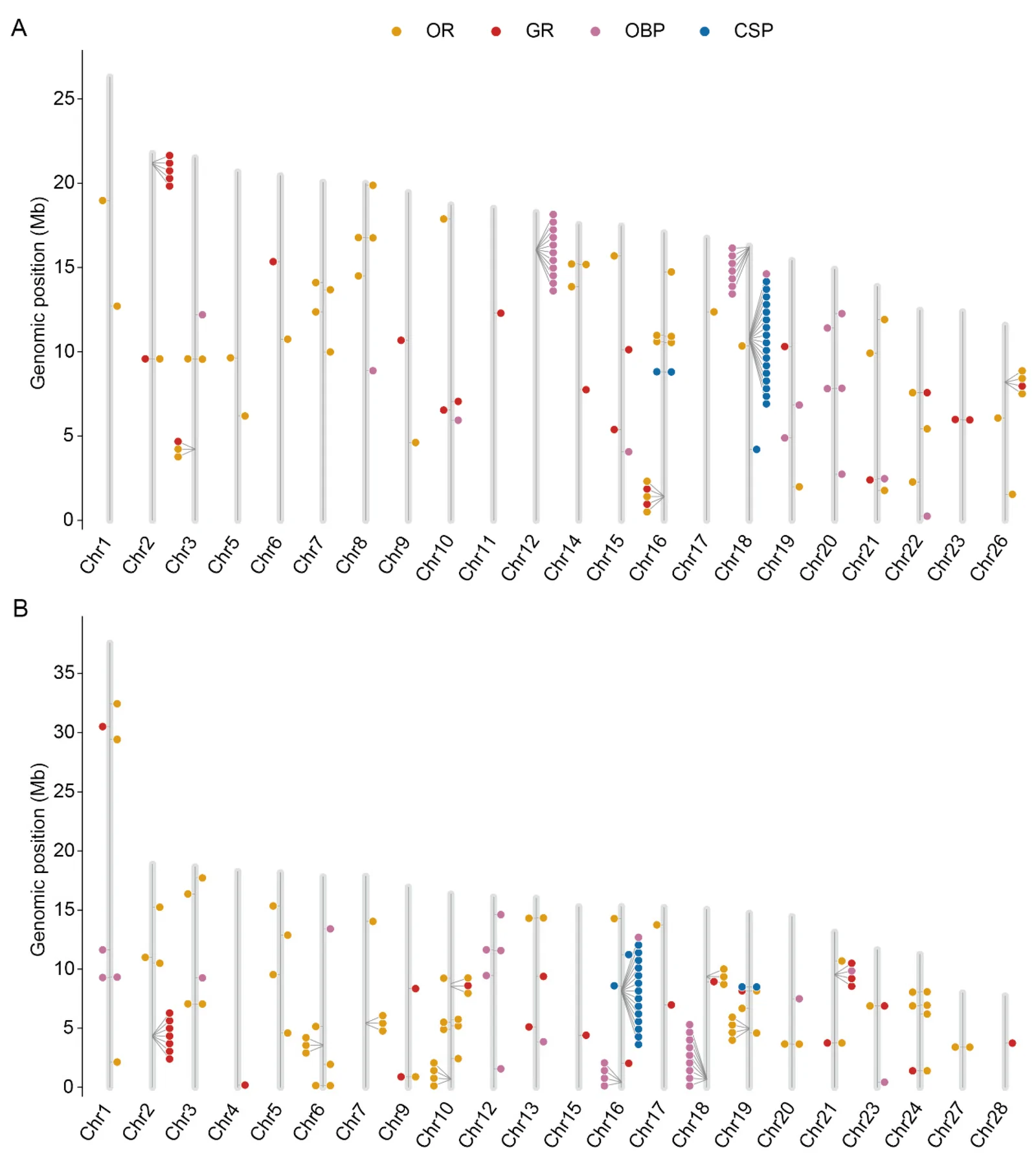
Chromosomal localization of chemosensory genes in two walnut pests: (**A**) *Conogethes punctiferalis*; (**B**) *Atrijuglans aristata*.

In *C. punctiferalis*, the average length of all OR genes was 398.13 aa, and only one gene (*CpunOR11*) lacked a complete ORF. Transmembrane helices were predicted in 44 genes (95.65%) (Supplementary Table S4). The average MW, pI, II, AI, and GRAVY values were 45.56 kDa, 7.98, 38.13, 103.17, and 0.31, respectively. The 46 OR genes were unevenly distributed across 17 chromosomes (Figure 2A and Figure S1A); among these, Chr16 harbored the highest number of OR genes (eight), while eight other chromosomes each contained only a single gene, and the single incomplete OR gene *CpunOR11* was located on Chr5. In *A. aristata*, all OR genes possessed complete ORFs, with amino acid lengths ranging from 94 to 882 aa (average 357.00 aa) (Supplementary Table S5). TMD prediction revealed that 59 genes (90.77%) contained 2 to 10 transmembrane helices, and these genes comprised both intact and fragmentary gene models.

The average MW, pI, II, AI, and GRAVY values of all genes were 41.23 kDa, 8.25, 40.28, 103.89, and 0.31, respectively. These OR genes were distributed across 18 chromosomes, with Chr10 containing the highest number (12 genes), among which four were arranged in tandem (Figure 2B and Figure S1B).

In *C. punctiferalis*, all GR genes possessed complete ORFs, with an average length of 374.91 aa, and encoded proteins containing 1–8 transmembrane helices (Supplementary Table S6). The average MW, pI, II, AI, and GRAVY values were 42.47 kDa, 8.41, 39.60, 110.62, and 0.36, respectively. These GR genes were distributed across 14 chromosomes, with six genes on Chr2 and only one or two genes on each of the remaining chromosomes (Figure 2A). In *A. aristata*, the GR genes ranged from 81 to 828 aa in length, with an average of 375.00 aa (Supplementary Table S7). Transmembrane helix prediction revealed that 24 genes (92.31%) contained 1–9 transmembrane helices. The average MW, pI, II, and AI were 42.89 kDa, 8.47, 37.75, and 110.33, respectively, and the GRAVY values ranged from -0.0085 to 0.5685, with an average of 0.33. Chr2 harbored the largest number of GR genes (seven), which were closely distributed on this chromosome (Figure 2B).

Compared with the longer OR and GR genes, the OBP genes in *A. aristata* and *C. punctiferalis* ranged from 133 to 375 aa (average 175.07 aa) and from 115 to 405 aa (average 190.47 aa), respectively (Supplementary Tables S8 and S9). Signal peptide prediction revealed that 24 (85.71%) OBP genes in *A. aristata* possessed N-terminal signal peptides of varying lengths, consistent with their role as secretory carrier proteins [51], while 21 (65.63%) genes in *C. punctiferalis* were predicted to contain signal peptides. The average MW, pI, II, and AI values for the OBPs in *A. aristata* were 19.94 kDa, 6.43, 40.91, and 81.99, respectively, while those in *C. punctiferalis* were 21.42 kDa, 6.61, 41.39, and 80.94. In addition, the majority of OBP genes in both species exhibited negative GRAVY values, consistent with the hydrophilic nature of the OBP family. In *C. punctiferalis*, more than one-third of the OBP genes were tandemly distributed on Chr12 (Figure 2A; Supplementary Table S8), and six additional genes were arranged in tandem on Chr18, whereas two other genes were dispersed but located in close proximity.

Although both OBPs and CSPs belong to the extracellular secretory protein family and share the ability to bind and transport odorant molecules during the initial stages of olfactory signal transduction, CSPs are generally shorter in amino acid length than OBPs [16,52,53]. In our study, the average lengths of CSP proteins in *A. aristata* and *C. punctiferalis* were 134.61 aa and 138.20 aa, with corresponding molecular weights of 15.33 kDa and 15.75 kDa, respectively (Supplementary Tables S10 and S11). In both species, all CSP proteins except one in each (*AariCSP12* and *CpunCSP15*) were predicted to contain N-terminal signal peptides longer than 10 amino acids. The pI, II, and AI values for the CSP family in *A. aristata* were 7.73, 39.25, and 85.04, respectively; in *C. punctiferalis*, these values were 7.47, 39.04, and 80.04. Consistent with their hydrophilic nature, the GRAVY values of the CSP family in both species were negative. The CSP families in both *C. punctiferalis* and *A. aristata* were distributed on only two chromosomes: Chr16 and Chr18 in *C. punctiferalis*, as well as Chr16 and Chr19 in *A. aristata* (Figure 2). Both species formed gene clusters on one chromosome—Chr18 in *C. punctiferalis* and Chr16 in *A. aristata*—while only two genes were located on the other chromosome.

Overall, the 121 candidate genes from the four families in *C. punctiferalis* were distributed across 22 chromosomes, while the 137 candidates in *A. aristata* were all located on 22 chromosomes. These genes were either tandemly arrayed or dispersed on chromosomes. Moreover, the number of chemosensory genes was not correlated with chromosome length (*p* > 0.05; Supplementary Figure S2). For example, although Chr1 was the longest in both species, it contained only two and seven chemosensory genes, respectively.

### 3.4. Phylogenetic Analysis of Chemosensory Genes

**Figure 3:**
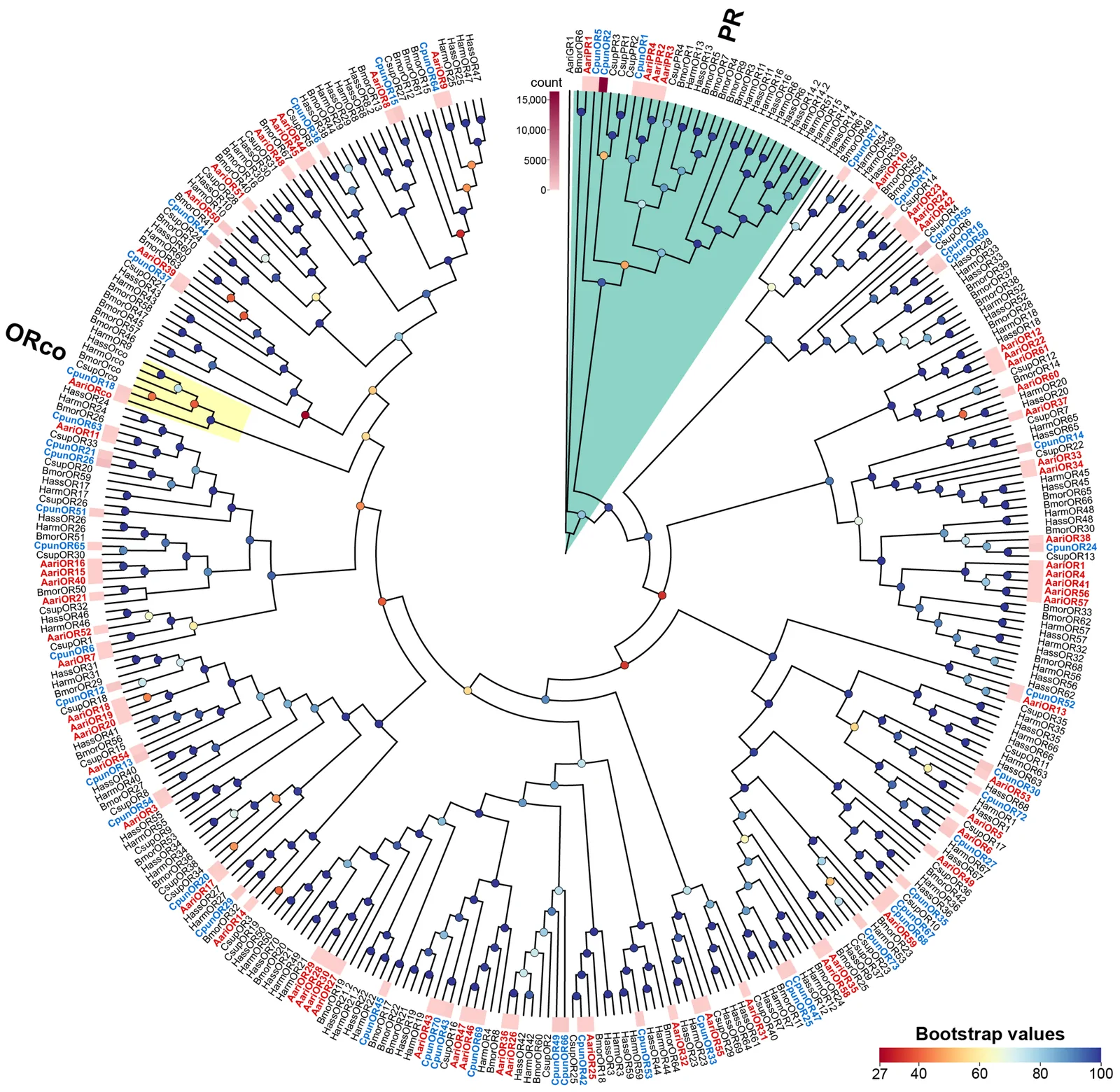
Maximum likelihood phylogenetic tree of candidate ORs from *Conogethes punctiferalis*, *Atrijuglans aristata*, and representative lepidopteran species, including *Chilo suppressalis* (Csup), *Bombyx mori* (Bmor), *Helicoverpa armigera* (Harm), and *Helicoverpa assulta* (Hass). Amino acid sequences of these species were obtained from ref. [55]. The ORco and PR clades are highlighted. Circle colors at each node represent bootstrap values.

Through phylogenetic analysis with other species, we identified a highly conserved co-receptor gene, *ORco*, in the genomes of both *C. punctiferalis* and *A. aristata*. The *ORco* genes from all species clustered together on the phylogenetic tree, reflecting their evolutionary conservation (Figure 3). In addition, three (*CpunOR1*, *CpunOR2*, and *CpunOR5*) and four (*AariPR1*–*AariPR4*) putative PR genes were identified in *C. punctiferalis* and *A. aristata*, respectively. These genes may play important roles in mate seeking during the adult stage [54]. The remaining OR genes were classified as conventional ORs. Notably, the additional OR genes in *A. aristata* compared with *C. punctiferalis* were also predominantly conventional ORs. RNA-seq expression profiling revealed that 42 of the 46 OR genes in *C. punctiferalis* and 14 of the 65 OR genes in *A. aristata* were transcriptionally active, indicating that these genes are likely functional at the transcript level.

**Figure 4:**
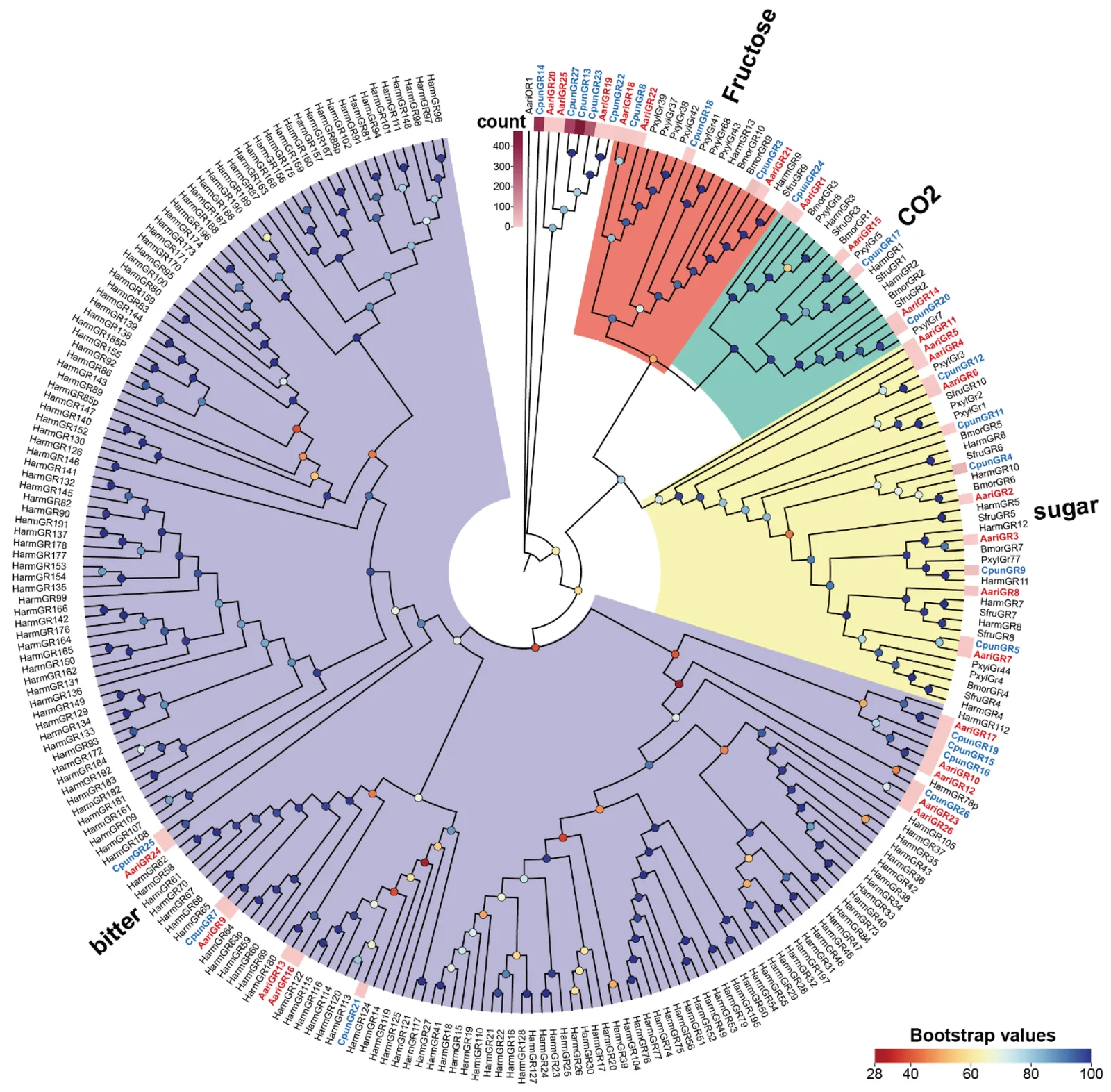
Maximum likelihood phylogenetic tree of candidate GRs from *Conogethes punctiferalis*, *Atrijuglans aristata*, and representative lepidopteran species. Amino acid sequences of other lepidopteran species were obtained from: *Helicoverpa armigera* (Harm) [56], *Spodoptera frugiperda* (Sfru) [23], *Plutella xylostella* (Pxyl) [57], and *Bombyx mori* (Bmor) [23].

Gustatory receptor genes were divided into four major clades on the phylogenetic tree: CO_2_ receptors, sugar receptors, fructose receptors, and bitter receptors (Figure 4). Among these, three genes in *C. punctiferalis* (*CpunGR3*, *CpunGR8*, and *CpunGR18*) and three in *A. aristata* (*AariGR18*, *AariGR21*, and *AariGR22*) were identified as putative fructose receptors, clustering with lepidopteran orthologs in a highly supported monophyletic group. In both species, three genes were assigned to the CO_2_ receptor clade: *CpunGR17*, *CpunGR20*, and *CpunGR24* in *C. punctiferalis*, and *AariGR1*, *AariGR14*, and *AariGR15* in *A. aristata*. Furthermore, five GR genes from *C. punctiferalis* (*CpunGR4*–*CpunGR5*, *CpunGR9*, and *CpunGR11*–*CpunGR12*) and eight from *A. aristata* (*AariGR2*–*AariGR8* and *AariGR11*) clustered with 24 other lepidopteran genes to form the sugar receptor clade. Among the four clades, the bitter receptor clade contained the largest number of genes, with seven and nine members identified in *C. punctiferalis* and *A. aristata*, respectively—a pattern consistent with findings in *Parafronurus youi* [53], *Phthorimaea operculella* [20], and *Phthorimaea absoluta* [20].

**Figure 5:**
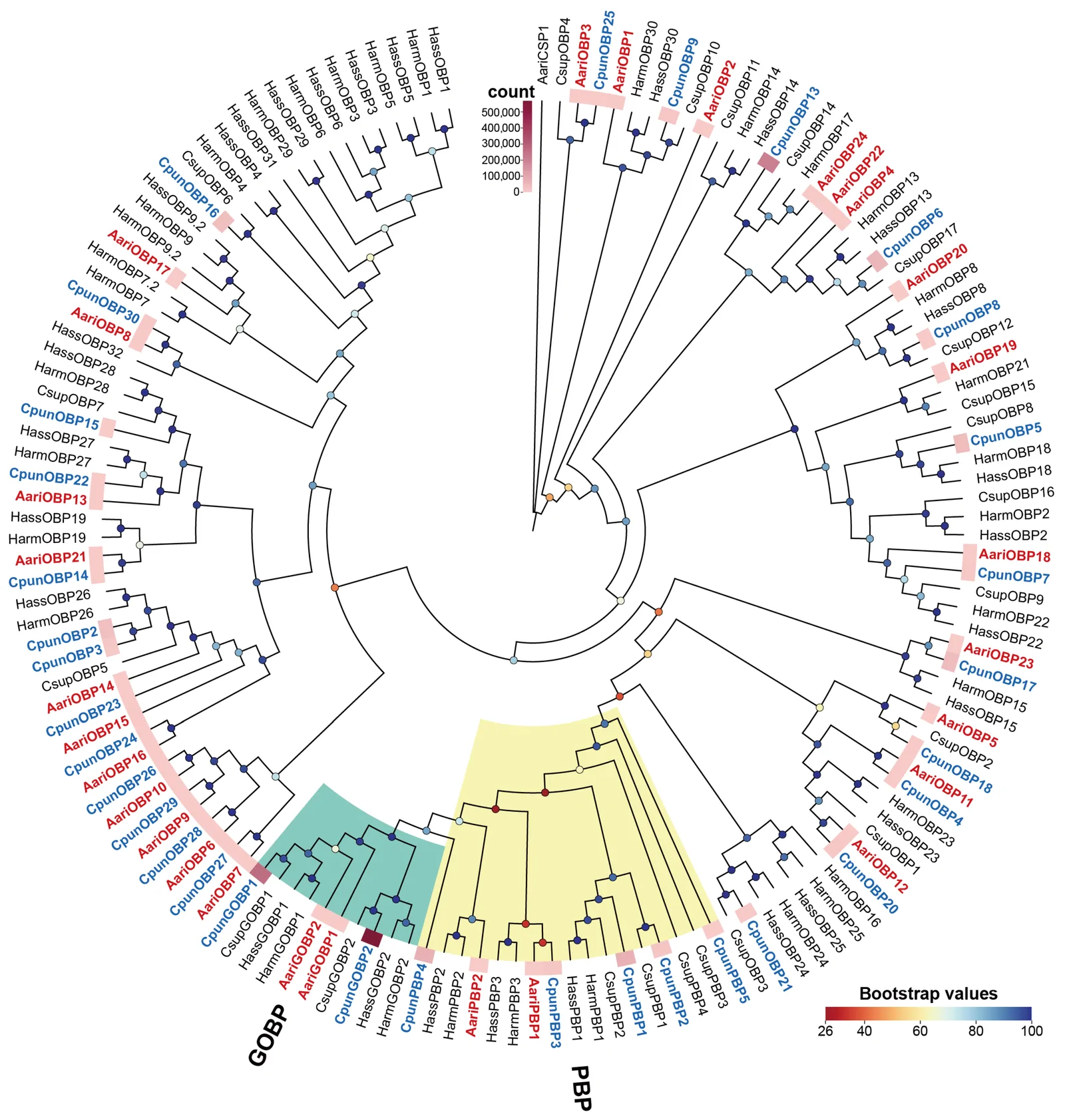
Maximum likelihood phylogenetic tree of candidate OBPs from *Conogethes punctiferalis*, *Atrijuglans aristata*, and representative lepidopteran species. Amino acid sequences of other lepidopteran species were obtained from *Chilo suppressalis* (Csup) [58], *Helicoverpa assulta* (Hass) [55], and *Helicoverpa armigera* (Harm) [59].

Through phylogenetic analysis, we identified five candidate PBP genes (*CpunPBP1–CpunPBP5*) and two GOBP genes (*CpunGOBP1*–*CpunGOBP2*) in *C. punctiferalis* (Figure 5). Notably, *CpunGOBP2* exhibited relatively high read coverage at the transcriptomic level, suggesting its elevated expression. In *A. aristata*, two genes (*AariPBP1*–*AariPBP2*) clustered within the PBP clade, while two others (*AariGOBP1*–*AariGOBP2*) were assigned to the GOBP clade. The PBP and GOBP clades formed sister groups, together constituting a monophyletic PBP/GOBP subfamily that was clearly distinct from the remaining conventional OBP sequences in the tree. Furthermore, transcriptome data revealed that over half of the OBP genes were expressed in both species.

**Figure 6:**
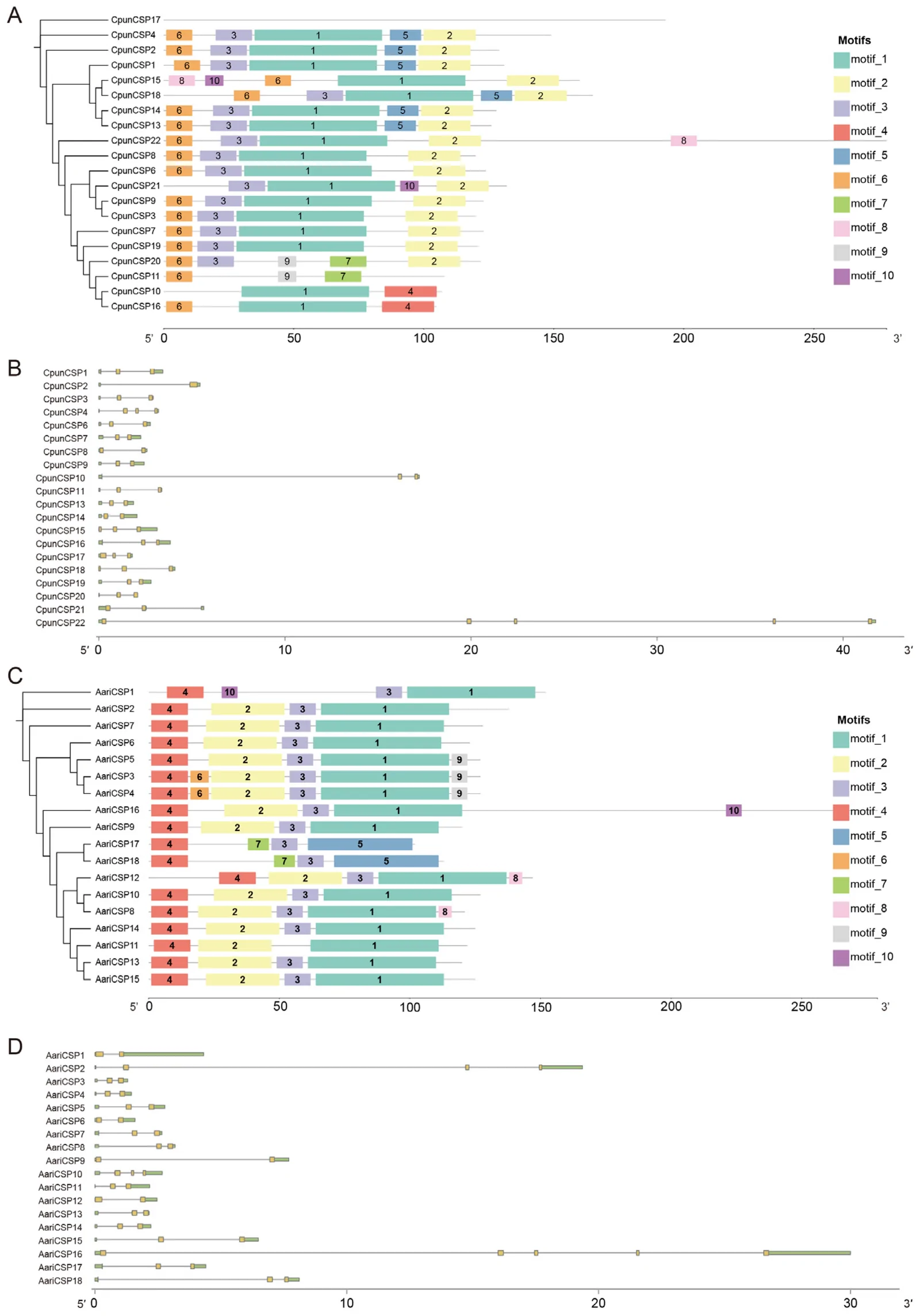
Phylogenetic relationships and conserved motifs, and gene structures of candidate CSPs in *Conogethes punctiferalis* (**A**,**B**) and *Atrijuglans aristata* (**C**,**D**).

Given that CSP proteins are structurally relatively conserved, we analyzed the conserved motifs and gene structures of the CSP family. Motifs 1, 2, 3, and 6 were present in the majority of CSP proteins in *C. punctiferalis*, representing the most conserved motifs (Figure 6A), whereas other motifs (4, 5, and 7–10) were present in fewer than half of the genes. Notably, *CpunCSP17* lacked any detectable motif and was the shortest CSP gene, at only 1800 bp (Supplementary Table S10). The majority of genes ranged from 2000 bp to 5000 bp in length (Figure 6B). *CpunCSP22* was the longest gene, containing two UTRs and five CDS regions; however, its relatively long intronic regions reduced the difference in amino acid length between this protein and other CSPs. In *A. aristata*, all CSP proteins contained detectable motifs, with motif 4 being the most conserved, present in all proteins (Figure 6C). In addition, motifs 1, 2, and 3 were retained in more than 80% of the family members. In terms of gene structure, *AariCSP1*, *AariCSP2*, and *AariCSP16* contained a 3’ UTR region exceeding 1500 bp, and more than 85% of the genes had a total gene length shorter than 9000 bp (Figure 6D).

## 4. Discussion

### 4.1. Genome Annotation Quality

Chemosensory perception plays a critical role in plant–insect interactions and behaviors such as mating and oviposition. Therefore, systematic identification of chemosensory gene families at the whole-genome level is a prerequisite for elucidating their molecular mechanisms and informing future pest management strategies. However, when comparing gene family numbers across insects with different dietary breadths, the use of different annotation methods can introduce substantial methodological biases [60,61,62]. Furthermore, the accuracy of gene family identification depends heavily on the quality of genome annotation [53,63,64]. To minimize such methodological biases, we re-annotated the genomes of *C. punctiferalis* and *A. aristata* using a standardized annotation pipeline. Compared with previous reports, our annotation results showed marked changes in gene numbers. In *C. punctiferalis*, 21,663 protein-coding genes were previously annotated, with an average gene length of 8420.9 bp [26]. In the present study, 13,236 genes were annotated, representing a reduction of 38.90%, whereas the average gene length increased substantially to 24,234.7 bp, with no substantial change in average CDS length (Table 1). Similarly, for *A. aristata*, the number of protein-coding genes decreased from 22,542 to 14,083 [25], a reduction of 37.53%. The marked reduction in gene number, together with the increase in average gene length, suggests that the EGAPx v0.2-alpha pipeline may consolidate fragmented gene models into complete and contiguous gene structures. Although EGAPx v0.2-alpha is an early-release version, this interpretation is supported by independent BUSCO and OMArk validations, both of which confirmed high completeness and consistency of the final gene sets [25,26]. Nevertheless, additional evidence (e.g., mapping of old and new gene models, gene length distributions, transcript support, annotation edit distance, and OMArk comparisons) is required to validate this conclusion.

### 4.2. Comparison of Chemosensory Gene Family Sizes Between the Two Species

In the *C. punctiferalis* genome, we identified 46 candidate ORs, 23 GRs, 32 OBPs, and 20 CSPs (Figure 1). These numbers are comparable to those of the Indian meal moth, *Plodia interpunctella*, a species belonging to the same superfamily, Pyraloidea [65], but are considerably lower than those of the notorious invasive pests, such as the cotton bollworm (*Helicoverpa armigera*) and the fall armyworm (*Spodoptera frugiperda*), particularly in terms of the GR family [24,44,60]. Notably, data type had a substantial impact on the identification results: only 10 GR genes were identified based on transcriptome data of *S. frugiperda* [23], whereas 221 GR genes were identified at the whole-genome level [24]. Similarly, transcriptome sequencing data of *C. punctiferalis* antennae identified 62 ORs and 10 GRs [27]. In the *A. aristata* genome, we identified 65 candidate ORs, 26 GRs, 28 OBPs, and 18 CSPs. In contrast, only one CSP and two OBPs were detected in transcriptome data derived from female pheromone glands and male genitalia [29]. Such discrepancies are attributable to tissue-specific expression or alternative splicing, which can generate multiple transcripts from a single gene [11,66,67]. Our findings thus reinforce the importance of using consistent methods when comparing gene families across species.

Although we cannot completely exclude the possibility that slight differences in RNA-seq support may have influenced the annotation results, the use of identical genome annotation and gene family identification pipelines for both species provides a reliable basis for examining the relationship between chemosensory gene family size. Previous studies have generally suggested that chemosensory gene families in polyphagous species, particularly the GR family, are more likely to undergo significant expansions that facilitate the detection of chemical signals from diverse host plants [68]. However, this pattern may not be universally applicable across all insect lineages. As shown in our study, *A. aristata* possesses more OR and GR genes than *C. punctiferalis*, with differences of 19 ORs (65 vs. 46) and 3 GRs (26 vs. 23) (Figure 1). Similar observations have also been reported in Papilionidae, where the specialist *Papilio memnon* has a greater number of both ORs and GRs compared to the generalist *Papilio glaucus* [69]. These findings suggest that the expansion of host plant range is not solely dependent on an increase in the number of chemosensory genes.

The evolution of chemosensory gene families generally follows the birth-and-death model [46]. Under this model, new genes arise through gene duplication events; during lineage divergence, some genes are retained in the genome as functional genes, whereas others are gradually lost due to the accumulation of deleterious mutations [70]. Consequently, chemosensory gene family size is shaped not only by dietary breadth but also by lineage-specific evolutionary histories. In this study, although both *A. aristata* (Stathmopodidae) and *C. punctiferalis* (Crambidae) infest walnut fruits, they are distantly related within Lepidoptera. The observed differences in chemosensory gene numbers between these two species may therefore partly reflect independent lineage-specific duplication and loss events.

Beyond lineage-specific evolutionary histories, ecological selection pressures represent another important factor shaping chemosensory gene family size. The insect chemosensory system is not solely dedicated to host plant recognition; it is also involved in the detection of non-host plants, symbiotic microorganisms, pheromones, and environmental chemical signals, as well as participating in various energy metabolic processes [69,71]. These diverse functions imply that the size of chemosensory gene families may be shaped by multiple ecological selection pressures. Notably, the adult *A. aristata* possesses enlarged hind legs with dense lanceolate scales on the tibiae and exhibits a characteristic leg-flicking behavior at rest [72]. Although it remains unclear whether this behavior is directly associated with chemoreception, this distinctive morphological trait suggests that *A. aristata* may occupy a specialized chemosensory ecological niche, which could also account for the larger chemosensory gene repertoire observed in this species.

It should be emphasized that the comparisons above focus primarily on differences in gene family size. Nevertheless, gene number does not necessarily equate to functional advantage. Whether the larger OR repertoire in *A. aristata* directly translates into an enhanced capacity to detect a broader spectrum of odorants, or whether these genes have undergone subfunctionalization, remains to be experimentally determined. Future studies integrating molecular docking, molecular dynamics simulations, and functional assays are required to systematically investigate the functional differences among individual genes in ligand recognition and to further evaluate the relationship between gene number and functional capacity.

### 4.3. Genomic Distribution and Phylogeny of Chemosensory Genes

In this study, chemosensory genes in both species were found to be either dispersed or clustered on chromosomes (Figure 2), a distribution pattern closely associated with gene duplication mechanisms [73]. Tandem duplication-generated paralogs are typically arranged in close proximity on chromosomes, share high sequence similarity, and may undergo subfunctionalization or neofunctionalization to adapt to new ecological demands [74,75,76]. These clustered genes may have undergone functional divergence in the recognition of different chemical ligands. In contrast, dispersed gene copies may have experienced longer periods of independent evolution, resulting in more pronounced functional divergence [77]. Collectively, these evolutionary mechanisms have shaped the diversity and functional adaptability of chemosensory gene families in insects.

Phylogenetic analysis revealed that both species possess conserved ORco and PR genes, with clustering patterns supporting their high sequence conservation within Lepidoptera. Notably, the additional OR genes in *A. aristata* were predominantly conventional ORs rather than PRs, suggesting that future studies should focus more on functional differences between the two species in the perception of plant volatiles, rather than pheromone components. Walnut fruits release complex volatile compounds, including monoterpenes such as β-phellandrene, sesquiterpenes, and the naphthoquinone juglone, some of which have been shown to influence host-seeking and oviposition behavior in walnut pests [78,79,80]. As a walnut specialist, *A. aristata may* require fine-scale discrimination of these host-specific chemical signals, and its larger OR repertoire might reflect an enhanced capacity to detect subtle differences within this narrower set of volatiles. Furthermore, within the OBP family, *C. punctiferalis* possessed five putative PBP genes, whereas *A. aristata* contained only two. The difference in PBP gene number may reflect a more complex pheromone communication system that facilitates successful mating across diverse host environments. However, these inferences remain to be validated by ligand-binding assays and functional gene analyses, which are essential for elucidating the evolutionary adaptation of chemosensory systems in insects with different feeding habits.

## 5. Conclusions

In this study, we performed genome annotation and chemosensory gene family identification using a standardized pipeline for two walnut pests with contrasting dietary breadths: the generalist *C. punctiferalis* and the specialist *A. aristata*. Genome annotation identified 13,236 and 14,083 protein-coding genes in the two species, with BUSCO completeness of 94.6% and 94.5%, respectively. Based on these annotations, we identified 121 candidate chemosensory genes (46 ORs, 23 GRs, 32 OBPs, and 20 CSPs) in *C. punctiferalis* and 137 (65 ORs, 26 GRs, 28 OBPs, and 18 CSPs) in *A. aristata*, with the latter possessing notably more OR candidates. Chromosomal distribution analysis further revealed that these genes were either tandemly arrayed or dispersed across the chromosomes, with members of each family clustering into conserved functional branches. Collectively, our findings provide comprehensive genomic resources for the two walnut pest species and a descriptive comparison of their chemosensory gene repertoires. These candidate genes not only serve as a resource for functional validation but also lay the groundwork for developing environmentally friendly walnut pest control strategies.

## Figures and Tables

**Table 1:** Statistics of the genome annotation for *Conogethes punctiferalis* and *Atrijuglans aristata*.

Features	*C. punctiferalis*	*A. aristata*
Number of protein-coding genes	13,236	14,083
Number of transcripts	19,272	22,461
Average gene length (bp)	24,234.7	21,951.9
Average exon per gene	8.00	7.72
Average exon length (bp)	324.9	369.1
Average full CDS length per longest transcript (bp)	1701.8	1645.8
Average CDS segment (exon) length (bp)	220.9	221.8
Average amino acid length of the longest transcript (aa)	566.3	547.6
Genes with complete ORF (%)	13,163 (99.45%)	13,996 (99.38%)
**BUSCO completeness**		
Complete BUSCOs (C)	1292 (94.6%)	1291 (94.5%)
Single-copy (S)	1279 (93.6%)	1241 (90.8%)
Duplicated (D)	13 (1.0%)	50 (3.7%)
Fragmented (F)	5 (0.4%)	10 (0.7%)
Missing (M)	70 (5.0%)	66 (4.8%)
**OMArk completeness**		
Single-copy	5818 (88.22%)	5549 (84.14%)
Duplicated	281 (4.26%)	574 (8.70%)
Missing	496 (7.52%)	472 (7.16%)
**OMArk consistency**		
Consistent lineage placements	11,880 (89.76%)	12,558 (89.17%)
Inconsistent lineage placements	568 (4.29%)	638 (4.53%)
Unknown	788 (5.95%)	887 (6.30%)
Contaminants	0	0

Bold entries represent categories that are further quantified by the detailed metrics listed below them.

## Data Availability

All relevant data have been deposited in the Figshare repository (https://doi.org/10.6084/m9.figshare.33256305, accessed on 25 August 2026), including genome annotation files, CDS and amino acid sequences, BLAST and HMM search results, analysis scripts, configuration files, seed sequences, command lines, alignments and trimmed alignments, tree files, MEME outputs, and all input files used to generate the figures. These data will be made publicly available immediately upon the acceptance of our manuscript.

## Supplementary Materials

The following supporting information can be downloaded at https://www.mdpi.com/article/10.3390/biology15171471/s1, Table S1. Summary of transcriptome sequencing data for Conogethes punctiferalis and Atrijuglans aristata. Table S2. Summary of BLAST and HMM detection results for chemosensory gene families. Table S3. Correspondence between transcriptome-derived OR candidates and genome-annotated genes in C. punctiferalis. Table S4. Classification and physicochemical characterization of odorant receptor genes in Conogethes punctiferalis. Table S5. Classification and physicochemical characterization of odorant receptor genes in Atrijuglans aristata. Table S6. Classification and physicochemical characterization of gustatory receptor genes in Conogethes punctiferalis. Table S7. Classification and physicochemical characterization of gustatory receptor genes in Atrijuglans aristata. Table S8. Classification and physicochemical characterization of odorant-binding protein genes in Conogethes punctiferalis. Table S9. Classification and physicochemical characterization of odorant-binding protein genes in Atrijuglans aristata. Table S10. Classification and physicochemical characterization of chemosensory protein genes in Conogethes punctiferalis. Table S11. Classification and physicochemical characterization of chemosensory protein genes in Atrijuglans aristata. Figure S1. Distribution of chemosensory genes across all chromosomes in the two walnut pests: (A) Conogethes punctiferalis; (B) Atrijuglans aristata. Figure S2. Correlation between chromosome length and number of chemosensory genes: (A) *Conogethes punctiferalis*; (B) *Atrijuglans aristata*.

## Abbreviations

The following abbreviations are used in this manuscript:

ORs	odorant receptors
GRs	gustatory receptors
OBPs	odorant-binding proteins
CSPs	chemosensory proteins
ORFs	Open Reading Frames
TMDs	transmembrane domains
MW	molecular weight
pI	isoelectric point
II	instability index
AI	aliphatic index
GRAVY	grand average of hydropathicity
