# Supplementary material for: Genomic Identification and Comparative Characterization of Chemosensory Genes in Two Walnut Pests
Source: Biology (Basel). 2026 Aug 31;15(17):1471. doi: 10.3390/biology15171471 (PMC13565414; doi:10.3390/biology15171471)
Supplement: Supplementary file 1 [file biology-15-01471-s001.zip › Supplementary_Figures_S1-S2.pdf]

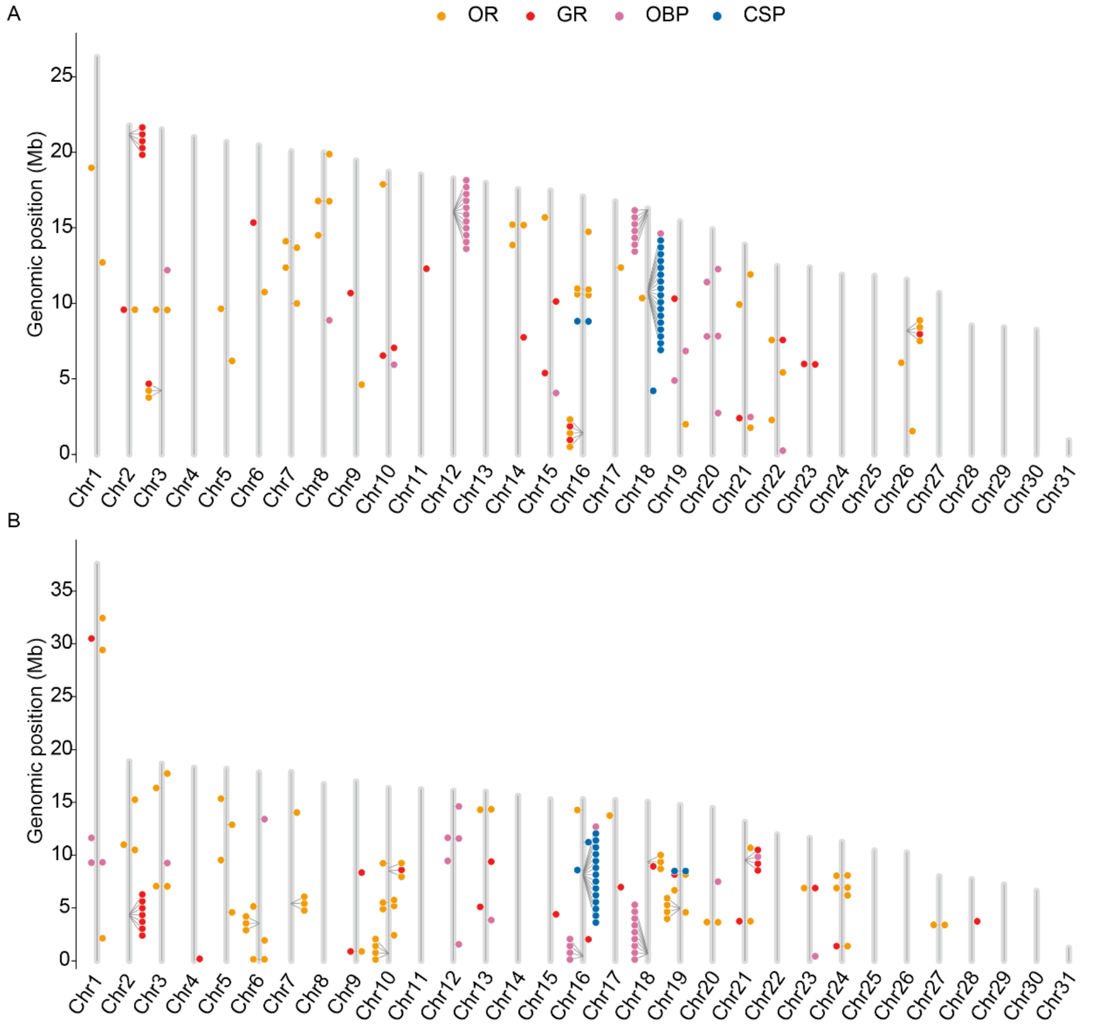

Figure S1. Distribution of chemosensory genes across all chromosomes in the two walnut pests. (A) *Conogethes punctiferalis* (B) *Atrijuglans aristata.*

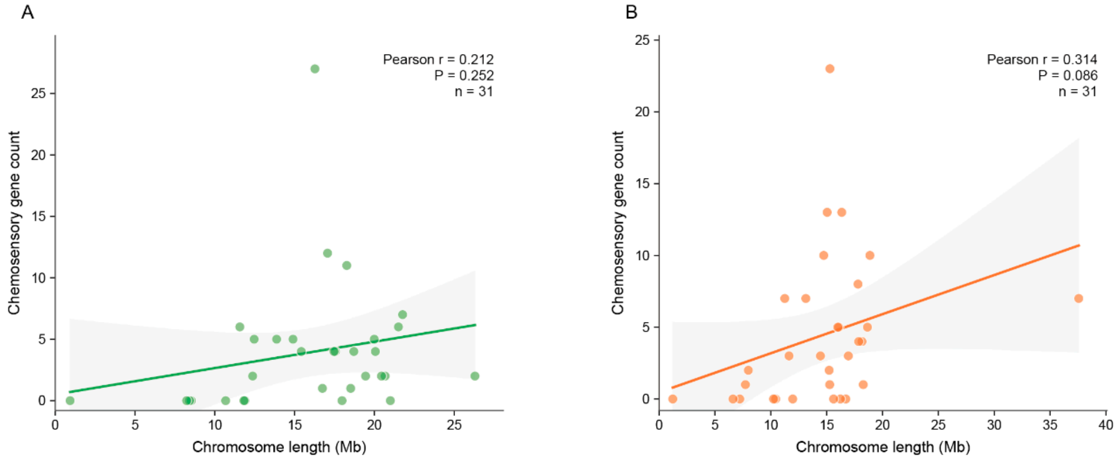

Figure S2. Correlation between chromosome length and number of chemosensory genes.

(A) *Conogethes punctiferalis* (B) *Atrijuglans aristata.*
